# Analog Lock-In Amplifier Design Using Subsampling for Accuracy Enhancement in GMI Sensor Applications

**DOI:** 10.3390/s23010057

**Published:** 2022-12-21

**Authors:** José M. Algueta-Miguel, J. Jesús Beato-López, Antonio J. López-Martín

**Affiliations:** 1 Institute of Smart Cities, Universidad Pública de Navarra (UPNA), Campus Arrosadia, 31006 Pamplona, Spain; 2 Departamento de Ciencias, Institute for Advanced Materials and Mathematics INAMAT2, Universidad Pública de Navarra (UPNA), Campus Arrosadia, 31006 Pamplona, Spain

**Keywords:** lock-in amplifier, phase-sensitive detector, GMI sensor, subsampling, sample-and-hold

## Abstract

A frequency downscaling technique for enhancing the accuracy of analog lock-in amplifier (LIA) architectures in giant magneto-impedance (GMI) sensor applications is presented in this paper. As a proof of concept, the proposed method is applied to two different LIA topologies using, respectively, analog and switching-based multiplication for phase-sensitive detection. Specifically, the operation frequency of both the input and the reference signals of the phase-sensitive detector (PSD) block of the LIA is reduced through a subsampling process using sample-and-hold (SH) circuits. A frequency downscaling from 200 kHz, which is the optimal operating frequency of the employed GMI sensor, to 1 kHz has been performed. In this way, the proposed technique exploits the inherent advantages of analog signal multiplication at low frequencies, while the principle of operation of the PSD remains unaltered. The circuits were assembled using discrete components, and the frequency downscaling proposal was experimentally validated by comparing the measurement accuracy with the equivalent conventional circuits. The experimental results revealed that the error in the signal magnitude measurements was reduced by a factor of 8 in the case of the analog multipliers and by a factor of 21 when a PSD based on switched multipliers was used. The error in-phase detection using a two-phase LIA was also reduced by more than 25%.

## 1. Introduction

The characterization of magnetic fields is fundamental in many relevant technological sectors, namely, automotive, transport, aero spatial, etc., [1,2]. In this scenario, different magnetic sensing principles have been proposed for the development of suitable magnetic sensors, such as giant magneto-resistive sensors [3,4], resonant coil sensors [5], fluxgate sensors [6,7], Hall sensors [8], spin valve sensors [9], superconducting quantum interference devices (SQUID) [10], etc. Among them, sensors based on the giant magneto-impedance effect (GMI) have been revealed as a powerful tool due to their faster response, smaller size, higher stability, lower cost, and especially their generally higher sensitivity during detection [2,11]. These features have enabled their use to spread in different fields, employing geometries and configurations such as amorphous wires, [12] ribbons [13], microwires [14], or thin films [15,16], among others. 

The GMI effect can be defined as the huge changes experienced by the high-frequency impedance, *Z = R + jX* (where *R* is the resistance and *X* the reactance) of a ferromagnetic conductor when it is exposed to a static DC magnetic field *H* [2,11]. The development of magnetic sensors based on the GMI effect has been gaining importance due to the growing necessity to tackle the detection of increasingly lower variations in the magnetic field value. This research effort demands the optimization of the device response during the detection procedure, a goal that can be accomplished, among other ways, through the enhancement of the synergetic coupling between the GMI sensing element and the signal conditioning circuitry. This circumstance turns the lock-in amplifier into one of the most suitable choices for signal detection in GMI-based applications.

A lock-in amplifier (LIA) is an electronic system used to recover very small AC signals in extremely noisy environments, even with noise and interference levels thousands of times larger than the amplitude of the target signal [17,18]. LIAs are also employed for precision measurement in scenarios with a good signal-to-noise ratio, but where extremely small amplitudes and/or phase variations need to be detected [17]. The core of the LIA is based on a phase-sensitive detector (PSD), conceived for detecting the amplitude and phase variations in a signal at a given frequency previously known. The LIA applications include a wide range of disciplines in applied science and technology, such as spectroscopy, biomedical measurements, complex impedance characterization, and photometry to name just a few [17]. During the last decades, the popularization of microprocessor and FPGA-based systems have led to the gradual substitution of traditional analog LIAs by digital implementations [4,18,19,20,21], mainly due to their robustness and programmability without compromising their key specifications at the expense of higher complexity and extra computing power. This digitalization tendency is induced, in part, by the difficulty of implementing high-performance PSD circuits based on analog multiplication. 

Analog multipliers are prone to generate distortion and DC offset due to their nonlinear operation [22,23,24]. The magnitude of this offset increases with input amplitude and operation frequency, potentially compromising the proper operation of the system. More concretely, output DC offsets may be a severe problem in lock-in circuits based on analog multipliers, since the offset will be indistinguishable from the useful DC value provided by the PSD. Although several techniques for offset cancelation in multipliers have been proposed in the literature [25,26], they are conceived for canceling not only offset but also any DC component at the output, which makes them unsuitable for PSD applications. A different approach for the DC stabilization of analog multipliers using chopper techniques has been presented in [24]. Although it preserves the DC input components, any useful information associated with the multiplier DC output will be lost as well. Hence, there is no effective solution for separating the useful DC value at the PSD output from the offset introduced by the analog multiplier, so its use in LIA design is limited to favorable conditions in which the generated offset is negligible, i.e., low operation frequencies. For instance, an analog LIA for phase measurement based on a PSD using the AD633 multiplier (Analog Devices) [27] has been presented in [28]. It provides high-resolution phase detection operating at 77 Hz. 

An alternative approach consists of implementing the multiplication function by means of switched multiplier schemes [7,29,30]. In this case, the sinusoidal reference signal is substituted by a ±1 square waveform of the same frequency. However, this PSD scheme leads to additional drawbacks: on the one hand, odd harmonics associated with the square waveform are introduced on the reference channel, which may induce misleading results if spectral components at these frequencies are present at the PSD input; on the other hand, the switched circuit may produce delay and jitter which may provoke synchronization problems at high frequencies [31,32].

As can be concluded from the above, the severity of both drawbacks linked to analog PSD design is strongly alleviated as the operation frequency decreases. In sensor-based applications, this frequency of operation is typically constrained by the sensor excitation frequency range. In particular, GMI amorphous wires and ribbons typically operate in the range of hundreds of kHz to a few MHz to maximize the GMI effect [11], while other GMI sensing elements such as amorphous microwires or thin films generally operate concretely at larger frequencies, from several tens of MHz to even GHz values [14,15,16]. In these latter cases, most of the related works in the literature employ expensive high-performance laboratory or computer-aided equipment for experimental measurements [14,15,16], due to the difficulty of designing low-cost, compact, and portable analog electronics at these frequencies. However, the operation frequency of GMI wires is suitable for the implementation of simple ad hoc lock-in systems based on analog PSDs, but, in contrast, the aforementioned problems associated with analog multiplication may begin to be significant when operating at hundreds of kHz.

In this context, a subsampling technique for frequency downscaling in analog LIA design using GMI sensors is proposed in this work. Specifically, both the output sensor and reference signals at the PSD inputs, initially at 200 kHz, are frequency-downscaled to 1 kHz to exploit the inherent advantages of signal multiplication at low frequencies. In this way, the functionality of the PSD remains unaffected if both input signals are scaled by the same factor, while the accuracy of the LIA is strongly enhanced. As proved later, the benefits of the proposed technique are evident both for conventional analog and switched multipliers. This frequency downscaling process is carried out by utilizing a subsampling process [33,34] employing sample-and-hold (SH) circuits [35]. 

Although the proposed technique has been applied to GMI sensors in this work, it is theoretically extensible to any LIA using an analog PSD. A practical limitation may arise from the growing complexity of implementing a precise SH block as the operating frequency increases. However, accurate SH circuits can be easily assembled using discrete components for working at frequencies of hundreds of kHz.

The paper is organized as follows: a brief review of the LIA fundamentals is provided in Section 2. In Section 3, the proposed subsampling technique for frequency downscaling in analog LIAs is presented. The complete experimental setup including the GMI wire, sensor interface, laboratory instrumentation, and the assembled LIA circuits is explained in Section 4. The experimental results are presented and discussed in Section 5, and finally, some conclusions are drawn in Section 6.

## 2. Fundamentals of LIAs

**Figure 1:**
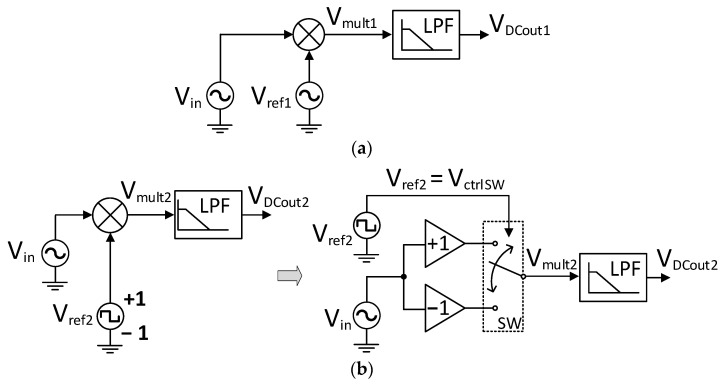
Basic block diagram of a phase-sensitive detector (PSD): (**a**) using conventional analog multiplication; and (**b**) multiplying by a ±1 square wave employing a switched multiplier.

An LIA can be understood as a measurement system for signal recovery operating on the PSD principle, also supported by amplification and processing stages [17]. The LIA approach requires, by definition, some previous knowledge about the signal that must be detected, and the parameters of the reference signal must be adjusted accordingly. The principle of operation of a PSD is shown in Figure 1a.

Let’s assume that the input signal *V_in_* is formed by the sum of the signal of interest *A_i_·sin*(*ω_i·_t* + *ϕ_i_*) and a generic signal *n*(*t*) that contains unwanted components at other frequencies. Using a sinusoidal reference signal *V_ref1_ = A_r_·sin*(*ω_r·_t + ϕ_r_*), whose frequency is precisely synchronized with the target signal (*ω_r_* = *ω_i_*), the multiplier output *V_mult1_* will be given by

(1) $$V_{mult1}=\frac{A_{i}\cdot A_{r}}{2}\cos\left(\phi_{r}-\phi_{i}\right)-\frac{A_{i}\cdot A_{r}}{2}\cos\left(2\omega_{i}t+\phi_{r}+\phi_{i}\right)+n\left(t\right)\cdot A_{r}\sin\left(\omega_{i}t+\phi_{r}\right)$$

Hence, the multiplier performs a synchronous detection that leads to an output voltage with a DC component corresponding to the first term of (1). This DC term can be straightforwardly obtained by using a very low-frequency low-pass filter (LPF), resulting in:

(2) $$V_{DCout1}=\frac{A_{i}\cdot A_{r}}{2}\cos\left(\phi_{r}-\phi_{i}\right)$$

Note from (2) that amplitude and phase variations cannot be detected at the same time by the PSD of Figure 1. On the one hand, variations in *ϕ_i_* can be precisely measured if the amplitude of the input signal A_i_ remains constant. In this case, the PSD output voltage will be proportional to cos(*ϕ_r_* − *ϕ_i_*). On the other hand, amplitude variations can be faithfully detected if a zero-phase difference between *V_ref_*_1_ and the signal of interest is forced, i.e., *ϕ_r_* = *ϕ_i_*. The implementation of the equivalent PSD employing switched multipliers is illustrated in Figure 1b. The sinusoidal reference signal *V_ref_*_1_ has been substituted by a ±1 square wave of the same frequency (*V_ref_*_2_), whose Fourier series is given by

(3) $$V_{ref2}=\frac{4}{\pi }\overset{\infty }{\underset{n=1}{\sum }}\frac{1}{2n-1}sin\left[\left(2n-1\right)\left(\omega_{r}t+\phi_{r}\right)\;\right]$$

In this case, assuming again that *ω_r_* = *ω_i_*, the following multiplier output *V_mult2_* is obtained:

(4) $$V_{mult2}=\frac{2A_{i}}{\pi }\cos\left(\phi_{r}-\phi_{i}\right)-\frac{2A_{i}}{\pi }\cos\left(2\omega_{i}t+\phi_{r}+\phi_{i}\right)+n\left(t\right)\cdot \frac{4}{\pi }\sin\left(\omega_{i}t+\phi_{r}\right)\;+\frac{4}{\pi }\left\{A_{i}sin\left(\omega_{i}t+\phi i\right)+n\left(t\right)\right\}\cdot \overset{\infty }{\underset{n=2}{\sum }}\frac{1}{2n-1}sin\left[\left(2n-1\right)\left(\omega_{r}t+\phi_{r}\right)\;\right]$$

Despite the complexity of (4), note that the final low-pass filter of the multiplier of Figure 1b will remove most of the terms as occurred with (2), just keeping the first DC term:

(5) $$V_{DCout2}=\frac{2A_{i}}{\pi }\cos\left(\phi_{r}-\phi_{i}\right)$$

Although Equation (5) is ideally similar to Equation (2), it must be considered that if n(t) contains spectral components at odd multiple frequencies of $\omega_{i},$ they will be multiplied by the corresponding harmonic of the square reference signal according to the last term of Equation (4). Hence, undesired DC terms will be added to Equation (5) and the accuracy of the PSD will be degraded. Moreover, another limitation may arise from the potential delay, t_d_, that the switch SW of Figure 1b may introduce. If this delay, t_d_, is low enough to become negligible against the period of the PSD input signal (t_d_ << 2π/$\omega_{i}$), then Equation (5) will remain unaffected in practice. However, if t_d_ provokes a significant phase-shifting on the reference signal, the effective value of $\phi_{r}$ will be modified, leading to a source of error in *V_DCout_*_2._

**Figure 2:**
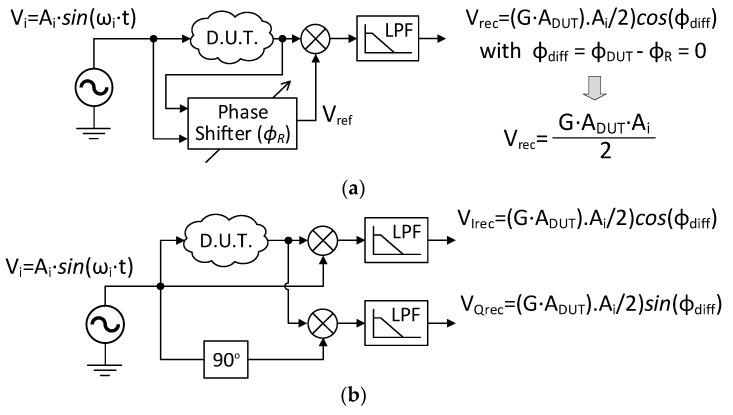
Scheme of typical lock-in amplifier implementations: (**a**) single-output lock-in amplifier; and (**b**) two-phase lock-in amplifier.

Once the PSD fundamentals have been addressed, a basic LIA architecture for amplitude detection is shown in Figure 2a. It operates as follows: a device-under-test is excited by an input sinusoidal signal *V_i_*. Although in some applications the signal of interest might be related to higher harmonics or intermodulation products [36,37], its own fundamental frequency is targeted in most of the cases, so the reference signal V_ref_ can be directly generated from the input signal. A phase-shifting block is included to ensure a phase matching between both PSD inputs so that the final DC output, $V_{rec}$, is proportional to the amplitude of the signal of interest. Specifically,

(6) $$V_{rec}=\frac{G\cdot A_{DUT}\cdot A_{i}}{2}$$

where *A_DUT_* is the amplitude at the frequency of interest at the device-under-test output, *A_i_* is the amplitude of the excitation signal, and *G* is a generic gain introduced by the filter, multiplier or amplifier blocks throughout the system.

An alternative approach providing simultaneous detection of amplitude and phase variations is shown in Figure 2b. This circuit incorporates two PSDs operating with in-phase and quadrature reference signals, so the corresponding output voltages *V_Irec_* and *V_Qrec_* can be understood as the cartesian representation of the recovered signal *V_rec_*. Therefore, its instantaneous amplitude and phase can be straightforwardly calculated as:

(7) $$\left|V_{rec}\right|=\sqrt{V_{Irec}^{2}+V_{Qrec}^{2}}$$

(8) $$\phi_{rec}=tan^{-1}\left(\frac{V_{Qrec}}{V_{Irec}}\right)$$

The performance of practical lock-in systems may also be limited by different tradeoffs between the parameters of the LIA, such as dynamic range, linearity, dynamic reserve, or output stability [17], which must be adapted to each final application. 

## 3. Proposed LIAs Using Subsampling for Frequency Downscaling

The proposed frequency downscaling technique based on subsampling and using SH circuits is explained in this Section. It has been conceived in general for analog LIA design, with the purpose of alleviating the aforementioned drawbacks associated with the PSD design at moderate and high frequencies. Nevertheless, the implementation presented in this work is focused on GMI sensor applications operating at frequencies of hundreds of kHz. Firstly, the linearity of analog multipliers is enhanced as the operation frequency decreases [22,32], while the output offset is strongly reduced. Secondly, the delay and jitter introduced by switched multipliers on the reference channel become negligible if the period of the signal of interest becomes large enough [31,32].

**Figure 3:**
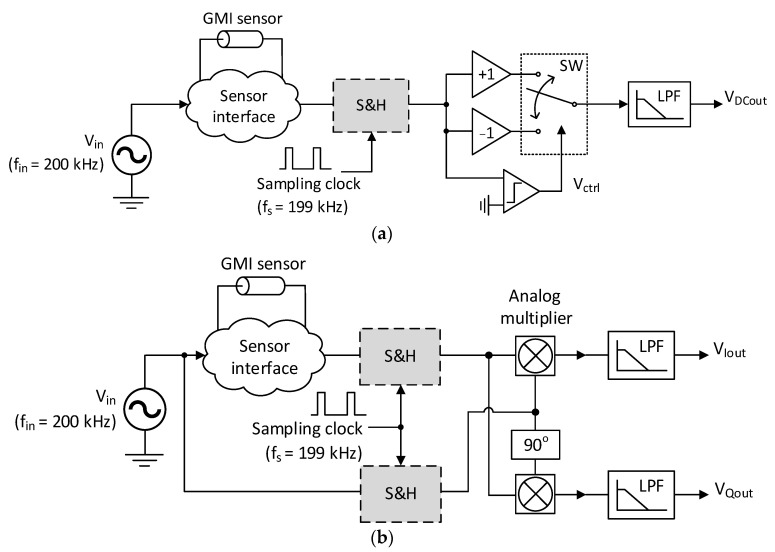
Block diagrams for the proposed LIA topologies using subsampling for frequency downscaling: (**a**) single-output LIA employing a switched multiplier; and (**b**) dual-output LIA using analog multipliers.

On this basis, an SH block has been included in the LIA topologies presented in Figure 3 for frequency reduction by means of a subsampling process. Both circuits are based on the general schemes of Figure 2 and have been adapted for detecting the fundamental harmonic at the output of a GMI sensor interface operating at 200 kHz. The PSD of the LIA in Figure 3a is based on the switched multiplication principle. In this case, the reference square wave is extracted from the sensor output signal itself through a comparator, leading to a simplified version of the scheme in Figure 2a. Specifically, the phase-shifting block has been excluded to avoid complicating the system beyond our purpose of validating the benefits of the proposed technique. Furthermore, the circuit of Figure 3b is a straightforward implementation of the scheme in Figure 2b using analog multipliers. Note that the SH blocks are properly placed for down-converting the operation frequency at both PSD inputs. A sampling frequency *f_s_* = 199 kHz has been employed so that the subsampled output signal is scaled down to *f_in_* − *f_s_* = 1 kHz. The fundamental of this subsampling process is explained in detail below.

### 3.1. Fundamentals of Subsampling

Subsampling can be defined as the sampling process of a bandpass signal using a frequency below the Nyquist frequency. Considering that the signal bandwidth (BW) is generally much lower than the center frequency *f_c_*, the subsampling may be carried out without aliasing between spectrum replicas [34]. Specifically, the spectrum of a sampled signal is given by

(9) $$X_{s}\left(f\right)=\left(1/T_{s}\right)\cdot \sum_{n=-\infty }^{\infty }X\left(f-nf_{s}\right)$$

where *X*(*f*) is the spectrum of the original signal, and *f_s_* and *T_s_* are, respectively, the sampling frequency and period. As explained in detail in [33], when a bandpass signal is subsampled at a frequency *f_s_* < *f_c_*, spectrum replicas are generated at −*m*·*f_s_* + *f_c_*, while mirrored replicas appear at (*m* + 1)·*f_s_* − *f_c_*, where *m* is an integer. A low-frequency replica of the original signal will be obtained at

(10) $$f_{L\;}=f_{c\;}-\;f_{s}\cdot floor\left(\frac{f_{c}}{f_{s}}\right)$$

This signal replica at *f_L_* can be extracted without aliasing if the following condition holds [33,38]:

(11) $$\frac{2\left(f_{c}-\frac{BW}{2}\right)}{n_{r}-1}<f_{s}<\frac{2\left(f_{c}+\frac{BW}{2}\right)}{n_{r}}$$

where *n_r_* is the number of replicas of the spectrum of the signal between 0 and *f_c_*-(*BW*/2). Note that at this point, the circuits in Figure 3 employ a sinusoidal excitation signal whose bandwidth ideally tends to zero, so the constraints stated in Equation (11) are relaxed in this application. As indicated in Figure 3, a frequency *f_s_* = 199 kHz has been chosen for sampling the signal at the sensor excitation frequency (200 kHz), so a low-frequency replica at *f_L_* =1 kHz would be obtained according to Equation (10). 

**Figure 4:**
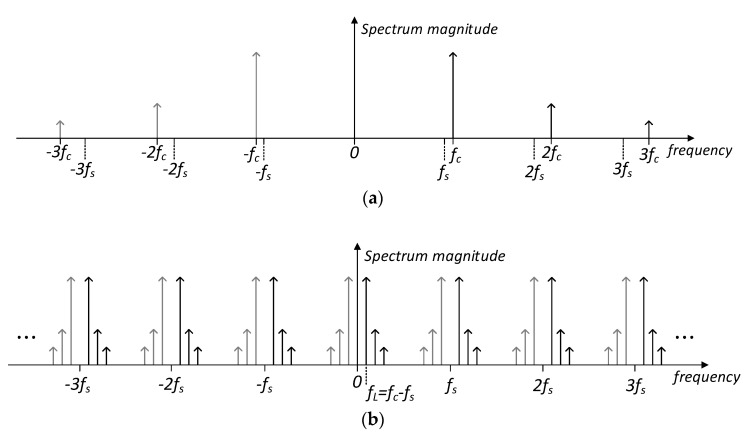

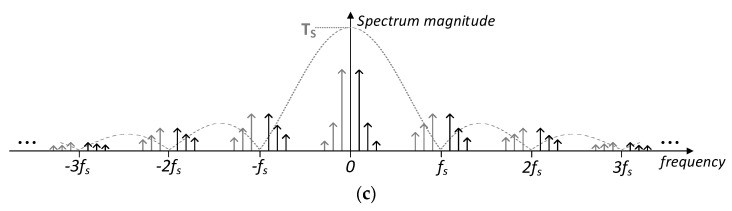
Illustration of the spectrum of a subsampled sinusoidal signal with a frequency *f_c_* and considering the presence of second and third-order harmonics. A sampling frequency *f_s_* slightly lower than *f_c_* is employed: (**a**) spectrum of the original signal; (**b**) spectrum of the ideally subsampled signal, according to Equation (9); and (**c**) spectrum of the subsampled signal by means of an SH circuit, which employs rectangular pulses with a duration *T_S_* according to Equations (12) and (13). Spectral lines are scaled by the value at their frequency of the *sinc* envelope shown by the dotted line.

To provide some insight, an illustrative example of the subsampling of a sinusoidal signal with a generic frequency *f_c_* is depicted in Figure 4. The spectrum of the original sinewave is plotted in Figure 4a, assuming the presence of second and third harmonics. A sampling frequency *f_s_* slightly lower than *f_c_* has been established. According to Equation (9), the spectrum shown in Figure 4b is obtained for the ideally subsampled signal. A low-frequency replica of the original signal is obtained at *f_L_ = f_c_* − *f_s_*, as stated in Equation (10). Note also that replicas of the second and third harmonics appear at frequencies 2·*f_L_* and 3·*f_L_*, respectively, so a whole downscaling of the spectrum in Figure 4a has been obtained. It is worth mentioning that a subsampling frequency of *f_s_* = 4·*f_c_/n_r_* (with *n_r_* an odd natural number) is recommended in communication systems for obtaining a signal replica at *f_s_*/4, thus relaxing the subsequent filter requirements for signal recovery [33,38]. However, a different criterion has been employed in the current application, since the benefits of the proposed downscaling technique enhance as *f_L_* decreases. Hence, the lower limit of *f_L_* is uniquely determined in practice by the electronics’ flicker noise, which may become dominant at frequencies below the kHz range [18], and by the possible requirements in terms of measurement speed, which depends on the target application.

### 3.2. Sample-and-Hold Considerations

**Figure 5:**
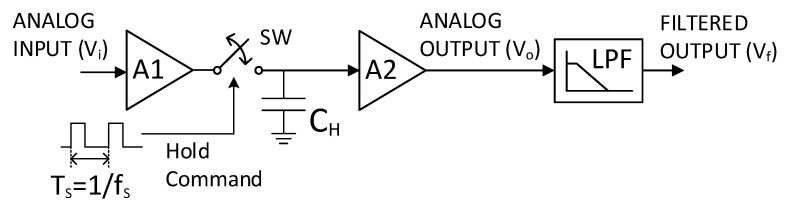
The basic scheme of an SH circuit.

**Figure 6:**
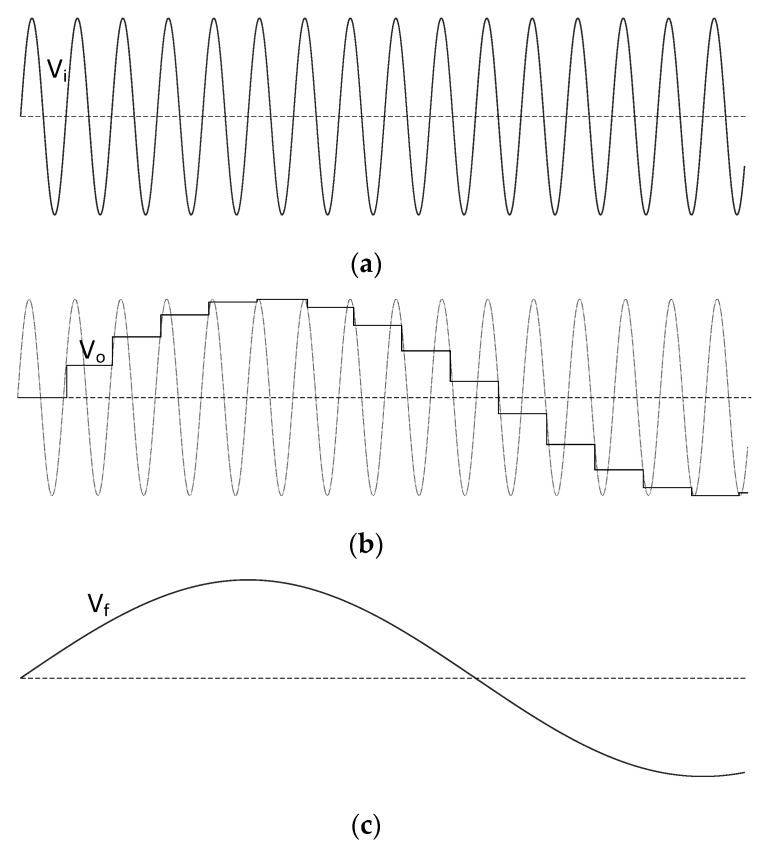
Illustration of the operation principle of the SH circuit in Figure 5 in the time domain. A sinusoidal signal is subsampled with a sampling frequency slightly lower than the sinewave fundamental frequency. The three voltages indicated in Figure 5 are plotted: (**a**) analog input of the SH, *V_i_*; (**b**) analog SH output *V_o_* with frequency downscaling; and (**c**) filtered output signal *V_f_*.

The previous analysis based on Equation (9) assumes that an ideal sampling is performed, i.e., the original signal is multiplied by a unit impulse train. However, when the basic SH architecture in Figure 5 is employed in practice, the original signal is multiplied by a train of rectangular pulses, as illustrated in Figure 6. The system operates as follows: an input amplifier buffers the input signal (Figure 6a) to charge the capacitor C_HOLD_. During the track mode (switch SW closed), the capacitor voltage follows the input signal, while in the hold mode (switch open) the sampled voltage is retained in C_HOLD_. A second amplifier with a very high input impedance is employed for driving the C_HOLD_ voltage, as well as preventing the capacitor from discharging prematurely [35]. As a result, a pulse-amplitude-modulated (PAM) signal with a pulse duration equal to the sampling period T_S_ is obtained [39], as depicted in Figure 6b. Under this assumption, Equation (9) becomes

(12) $$X_{S}\left(f\right)=\left(1/T_{s}\right)\cdot \sum_{n=-\infty }^{\infty }X\left(f-nf_{s}\right)\cdot H\left(f\right)$$

where *H*(*f*) is the Fourier transform of the rectangular pulse shape, in turn, given by

(13) $$H\left(f\right)=T_{s}\cdot sinc\left(fT_{s}\right)e^{-j\pi fT_{s}}$$

Returning to the example of Figure 4, the spectrum in Figure 4c corresponds to the subsampling process of the original signal using rectangular pulses with a duration of *T_s_*. Note from Equation (12) that the multiplication in the frequency domain of an ideally sampled signal by a sinc shape will distort the original spectrum proportionally to its bandwidth. However, in the particular case of a sinusoidal signal, the sinc multiplication just introduces an attenuation *T_s_*·|*sinc*(*f_L_*·*T_s_*)| at the fundamental frequency, and even the relative magnitude ratio with respect to the second and third harmonics (further attenuated) is increased, as can be observed in Figure 4c. Anyway, both effects become negligible if the ratio *f_c_*/*f_L_* is high enough.

## 4. Complete Experimental Setup

**Figure 7:**
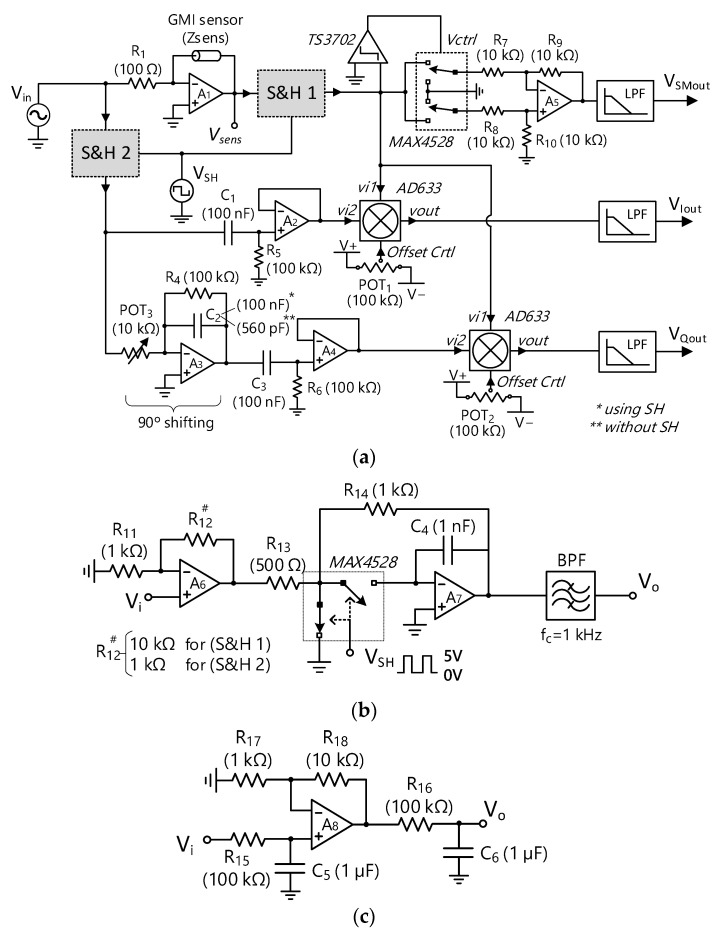
Complete schematic of the proposed system: (**a**) circuit containing the GMI sensor interface together with both proposed LIA architectures; (**b**) schematic of the SH; and (**c**) schematic of the low-pass filter at the LIA outputs.

The implementation of the LIAs in Figure 3 together with the experimental setup are addressed in detail in this Section. The complete schematic of the final circuit is shown in Figure 7. It comprises the GMI sensor, the electronic interface, and both LIA architectures. The LIAs have been tested with and without the SH blocks to confirm the benefits of the proposed frequency downscaling technique. A dual-supply voltage of ±5 V provided by an Agilent E3630A DC power supply was employed. The input voltages in Figure 7, V_in_ (sensor excitation signal) and V_SH_ (control signal of the SH switches) were produced by an Agilent 33,522A waveform generator. A USB oscilloscope, Digilent Analog Discovery 2, was employed to capture both the voltage signal at the output of the GMI sensor (V_sens_) and the DC output voltages of the proposed LIAs (V_SMout_, V_Iout,_ and V_Qout_). The circuit was assembled on a breadboard and the FET-input operational amplifier AD823 from Analog Devices [40] was used in all cases in Figure 7.

### 4.1. GMI Sensor and Electronic Interface

**Figure 8:**
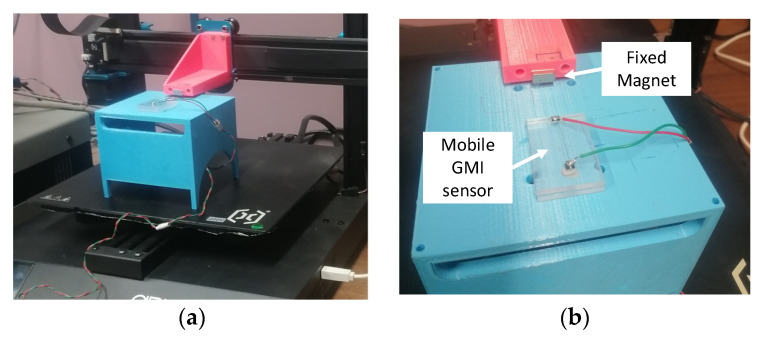
Photographs of the GMI sensor experimental setup: (**a**) general view of the sensor and magnet installation on the 3D printer motor; and (**b**) detail of the fixed neodymium magnet and the mobile GMI sensor.

An amorphous Co_66_Fe_2_Si_13_B_15_Cr_4_ wire (3 cm in length, and 90 μm in diameter) obtained by the in-rotating-water quenching technique [11] was employed as a GMI sensing element for testing the proposed LIAs. The wire was excited under optimal conditions for maximizing the GMI effect (15 mA_pp_ at 200 kHz). The transimpedance amplifier shown in the upper left corner of Figure 7 was employed for ensuring a constant current V_in_/R_1_ flowing through the sensor [41], where the sinusoidal input voltage V_in_ had an amplitude of 1.5 V_pp_ at 200 kHz. Under these conditions, the sensing wire impedance variations were analyzed under the effect of a magnetic field generated by a neodymium magnet. For the sake of accuracy and reproducibility, an adapted commercial 3D printer motor (Artillery Sidewinder x1) controlled by LabView was used to precisely vary the relative distance, x, between the sensor and the magnet. Specifically, the GMI sensor was placed on the mobile surface of the device while the magnet remained fixed. As a result of *x* diminution, the mean magnetic fields acting on the GMI sensing element increased, resulting in a constant impedance decrease [11]. Concretely, a maximum relative change in the impedance of around 75% was achieved within the applied magnetic field interval, i.e., 10 kA/m to 250 A/m. Two photographs of the setup are shown in Figure 8. 

### 4.2. Sample-and-Hold Schematic

The schematic of the blocks SH1 and SH2 in Figure 7a is depicted in Figure 7b. It has been developed from the basic SH scheme of Figure 5 including some extra improvements. On one hand, the two complementary switches (chip MAX4528 from Maxim Integrated [42]) provide a constant input impedance that prevents the input signal from coupling to the output during the hold period. On the other hand, the DC errors introduced by the switches are minimized by the feedback loop [35]. Moreover, the hold-capacitor C_4_ is also placed in the feedback path of the amplifier *A*_7_, so that the switching block always sees a virtual ground. In this way, the charge removed from the negative input of *A*_7_ by the parasitic capacitances of the switches remains constant regardless of the voltage at the output of *A*_7_ [32]. Finally, a 4th-order Butterworth bandpass filter was chosen for the output filtering.

The voltage V_SH_ controlling the sampling and holding periods was a 199 kHz square wave. The signal V_SH_ swung between 0 and 5 V with a duty cycle of 10%, i.e., the tracking period had a duration of 0.1·*T_s_* and the holding period was 0.9·*T_s_*. Note that the duration of the track mode must be large enough to allow the capacitor voltage to follow the input signal accurately. In the same way, the settling time must be also considered after switching to the hold mode, since the output voltage takes some time to settle within a specified error margin [32]. Nevertheless, the previous considerations are not a severe constraint in the proposed subsampling approach, since a very low sampling rate was employed, and consequently (since f_s_ is very close to *f_c_*) the voltage gap between consecutive samples was generally small. 

### 4.3. Considerations for LIA Implementation

The ±1 switched multiplier of the single-output LIA in Figure 3a was implemented in Figure 7 by using the phase-reversal analog switch MAX4528 [42]. Switching between the positive and negative inputs of the operational-amplifier-based subtractor (formed by *A_5_* and resistors *R*_7_–*R*_10_) alternates the sign of the amplifier gain. One chip 4116R-1-103FLF of integrated thick film 10 kΩ matched resistors was used for implementing *R*_7_–*R*_10_ since a high-resistance matching was required to obtain a precise ±1 gain. 

Regarding the two-output LIA in Figure 3b, the integrated circuit AD633 from Analog Devices [27] was chosen to perform the analog multiplication. As mentioned in the Introduction Section, DC offsets at the multiplier inputs may seriously degrade the quality of the signal detection, as the corresponding output DC values would be indistinguishable from the readout of the signal of interest. For this reason, two AC-coupled buffers (amplifiers *A*_2_ and *A*_4_) were placed at the multiplier inputs. Moreover, the voltages at the offset-control pins of both AD633 chips were adjusted by the potentiometers *POT*_1_ and *POT*_2_ to ensure a zero-voltage output when the inputs were grounded. The integrator circuit in the bottom left corner of Figure 7a was used for introducing the 90^o^ phase-shifting on the quadrature branch of the LIA. Different values for the potentiometer *POT*_3_ and the capacitor *C*_2_ were chosen for each operation mode of the circuit, i.e., with and without SH blocks, since the pole of the circuit at *ω_p_* = 1/(*C*_2_*·R*_4_) must be adapted to guarantee the integration functionality at each operation frequency (200 kHz and 1 kHz, respectively). The potentiometer *POT*_3_ was adjusted in each case to obtain the same amplitude in both the in-phase and quadrature reference signals.

Finally, the schematic of the output LPF blocks in Figure 7a is shown in Figure 7c. It consists of two RC sections with a non-inverting gain in between. The lower the LPF cutoff frequency, the better the recovery accuracy and the noise rejection of the LIA, which may lead to a challenging task in the design of fully integrated LIAs where the size of capacitances is strongly constrained [43]. However, the use of large RC time constants is not a limitation in discrete systems (or integrated systems with external capacitors), so the tradeoff between precision and measurement speed is the only consideration. In this case, 100 kΩ resistors and capacitors of 1µF were employed for the 2nd order LPF implementation.

## 5. Measurement Results

The voltage V_sens_ at the output of the sensor interface in Figure 7a was monitored by the digital oscilloscope. The magnitude of the fundamental harmonic was accurately calculated and averaged using the FFT function of the device software, while the phase variations were extracted from the time domain signal employing the same device. Both parameters were taken as references for evaluating the precision of the proposed LIAs. The LIA topology of Figure 3 (implemented according to Figure 7) was tested with and without the SH blocks, i.e., using frequency downscaling at the PSD inputs (to 1 kHz), and with the conventional PSD (at 200 kHz). The distance between the neodymium magnet and the GMI sensor was varied from 50 mm to 12.5 mm in steps of 2.5 mm.

**Figure 9:**
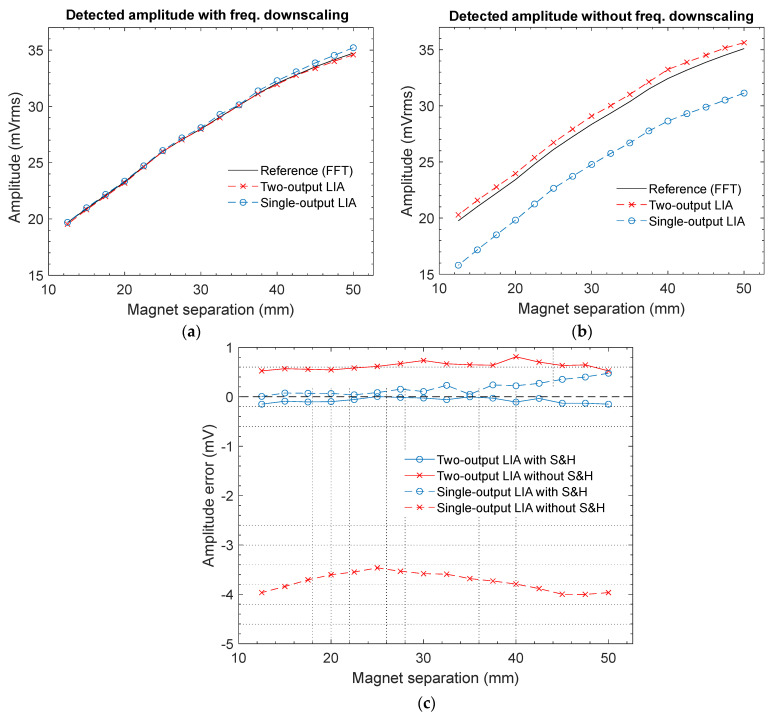
Amplitude measurements of the LIAs compared with the reference value provided by the FFT function of the digital oscilloscope, versus separation between GMI sensor and magnet: (**a**) amplitude detected by the proposed LIAs using SH blocks for frequency downscaling; (**b**) amplitude detected by the conventional LIAs without frequency downscaling; and (**c**) amplitude error for both LIA architectures with and without frequency downscaling.

The measurement results of the amplitude of the fundamental harmonic of V_sens_ (see Figure 7a) are plotted in Figure 9. In Figure 9a, the black solid line corresponds to the reference value of V_sens_ provided by the FFT function at 200 kHz, while the outputs of both LIAs using frequency downscaling are drawn in blue (single-output LIA) and red (two-output LIA) dashed lines, respectively. Note that the curves of both LIAs have been plotted with a scale factor such that the voltage swing between maximum and minimum values is the same as for the reference curve. In this way, a precise comparison in terms of offset can be performed. The same procedure is used in Figure 9b for the outputs of the conventional LIAs without frequency reduction. To provide more insight, the LIAs measurement error with respect to the FFT reference values is shown in Figure 9c for all the previous cases. Regarding the two-output LIA, the signal magnitude was externally calculated from the two captured output voltages (V_Iout_ and V_Qout_) according to Equation (7). Note that the error introduced by the analog multipliers was considerably reduced when frequency-downscaled input signals were employed. Specifically, the mean voltage deviation throughout the experiment decreased from 628.1 µV to 74.9 µV, which corresponded to a reduction factor of 8.39. The benefits of the proposed technique are even more noticeable in the case of the single-output LIA, in which the delay of the switches seriously deteriorated the accuracy of the PSD when the conventional topology was used. In this case, the effect of the switch delay was strongly alleviated thanks to the frequency downscaling, reducing the mean deviation of the voltage V_SMout_ in Figure 7a by a factor of 21.15, i.e., from 3.743 mV to 177 µV.

**Figure 10:**
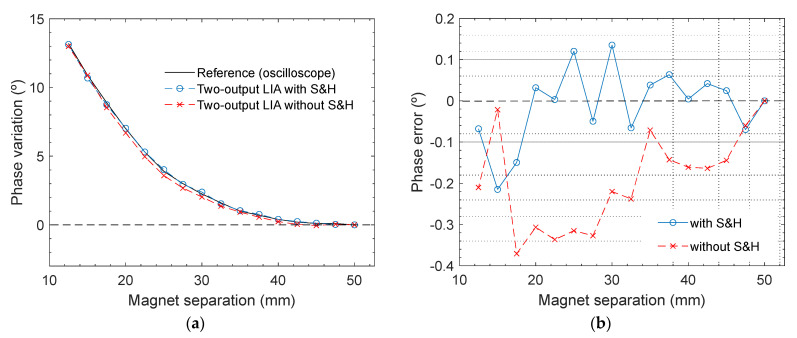
Phase variation measurements of the two-output LIAs compared with the reference value provided by the digital oscilloscope, versus the separation between the GMI sensor and magnet. The phase value with a magnet separation of 50 mm was set as the reference (0^o^): (**a**) representation of the phase variation with and without frequency downscaling; and (**b**) phase error.

Furthermore, the effect of the proposed technique on the phase response of the two-output LIAs (scheme in Figure 3b and outputs V_Iout_ and V_Qout_ in Figure 7a) were studied. In this case, a specific point needed to be established as a phase reference value (0°), so phase variations could be measured and compared. A sensor position 50 mm away from the magnet was arbitrarily set as the reference. From this point, the phase variation was monitored as the magnet and sensor approach, leading to the results plotted in Figure 10a. The reference black solid line was obtained by the phase function of the digital oscilloscope, which calculated the phase difference between V_sens_ in Figure 7a and the input signal V_in_, which served as a stable reference sinewave. A digital bandpass filtering centered at 200 kHz was also performed over both signals in order to attenuate possible higher-order harmonics that might have degraded the measurement accuracy. On the other hand, the blue and red dashed lines were obtained by applying Equation (8) to the LIA output voltages (calculations are performed externally), with and without employing the proposed technique. The representation of the phase errors is provided in Figure 10b.

The benefits of the proposed technique concerning phase measurement accuracy are not as significant as for the magnitude, as can be seen in Figure 10. This result was expected since if the same DC offset is added to both LIA output voltages, V_Iout_ and V_Qout_, the error in the tan^−1^(V_Qout_/V_Iout_) operation may be partially compensated. In fact, this error will be ideally zero if both in-phase and quadrature components have the same value initially. 

Nevertheless, the proposed technique also had a positive effect on the precision of the phase variations measurement. Note that the mean errors obtained from both lines in Figure 10b were conditioned by the arbitrary choice of the reference position (phase = 0°). For the sake of reliability, the mean phase deviation was calculated N times for N different reference points from the data in Figure 10, where N = 16 is the total number of points for each curve. These N mean values were subsequently averaged, leading to final mean error values of 0.130° for the conventional circuit and 0.097° for the circuit using frequency downscaling. Hence, the proposed technique reduced the phase measurement error by >25% compared to the conventional lock-in topology.

## 6. Conclusions

A subsampling technique for downscaling the signal frequency at the PSD inputs in analog LIA design, using analog and switched multiplication, respectively, has been proposed in this paper. Two LIA architectures conceived for GMI sensor applications operating at several hundreds of kHz have been assembled using discrete components on a breadboard. The accuracy of both LIA topologies using the proposed technique was compared with their conventional versions, obtaining a strong precision enhancement. In particular, the accuracy of the signal magnitude measurements increased by a factor of 8.39 when analog multipliers were employed, while an improvement factor of 21.15 was obtained when a PSD based on switched multiplication was used. Moreover, the error in-phase measurement using a two-phase LIA topology was also reduced by >25%.

## Data Availability

The data presented in this study are available on request from the corresponding author.
